# Supplementary material for: Nematic structures contribute to robust zygotic polarization in C. elegans
Source: PLoS Comput Biol. 2026 Sep 23;22(9):e1014792. doi: 10.1371/journal.pcbi.1014792 (PMC13630317; doi:10.1371/journal.pcbi.1014792)
Supplement: Supplementary file 1 [file pcbi.1014792.s001.pdf]

Supplementary Information

1 Supplementary Text

Text A: DCM force balance

Here, we provide the details of the DCM, based on Cuvelier et al. [1]. Cells behave as pressurized liquid "bubbles" with surface tension and internal pressure. The actomyosin cortex of a cell is represented by a viscous shell which is approximated by a triangulated surface mesh with effective thickness t_c, and nodal positions $\mathbf{x}_i$. In the following sections we will discuss different force contributions and their implementation.

Surface tension

Isotropic tension generated in the cortex results from actomyosin contractility, which is represented as an effective surface tension γ in the shell model. Using the derivation of Fedosov et al. [2], the direct force contribution from isotropic surface tension γ of node i is given by

$$\mathbf{F}_i^{\text{act}} = \frac{\gamma}{2}(\mathbf{x}_k - \mathbf{x}_j) \times \hat{\mathbf{n}}_A, \tag{1}$$

where i, j, k are nodes of a single triangle $A = (ijk)$. A small local area modulus K_A additionally penalizes deviations of individual triangle areas from their reference values, primarily for numerical stability.

Internal pressure

Since the cytoplasm is assumed to be incompressible, active volume control is implemented via a cytoplasmic pressure. We assume the equilibrium volume V_α^* of a cell α is constant and implemented a proportional-integral (PI) pressure controller to enforce this. The cytoplasmic pressure $P_\alpha^{\text{cyt}}(t)$ due to the volume controller is then estimated as

$$P_\alpha^{\text{cyt}}(t) = -K_V \epsilon_\alpha(t) - \frac{K_V}{T_I} \int_0^t \mathrm{d}s \; \epsilon_\alpha(s), \tag{2}$$

for a cell with volumetric strain $\epsilon_\alpha(t) = (V_\alpha(t) - V_\alpha^*)/V_\alpha^*$. T_I is typically chosen as one order of magnitude larger than the time-resolution with which the system is updated. The total pressure acting on a node

$$P_i(t) = \frac{2\gamma}{R_\alpha^{\text{cell}}} + P_\alpha^{\text{cyt}}(t),$$

includes an additional offset pressure $2\gamma/R_\alpha^{\text{cell}}$ to balance the actomyosin contractility in the cortex. This ensures cells are in mechanical equilibrium at the start of the simulations. The force acting on node i due to nodal pressure $P_i(t)$, is obtained by integrating over the node associated oriented Voronoi area S_i. Given the pressure is constant over the cell surface, the force is simply given by

$$\mathbf{F}_i^{\text{cyt}}(t) = \mathbf{S}_i \, P_i(t). \tag{3}$$

Medium damping

A general drag force

$$\mathbf{F}_i^{\text{drag}} = -\Gamma_{ii}^{\text{f}} \cdot \mathbf{v}_i,$$

where

$$\Gamma_{ii}^{\text{f}} = \frac{3\eta^{\text{f}}}{2R^{\text{cell}}} S_i \mathbb{I}, \tag{4}$$

is included to account for the liquid drag between the cells and their medium due to the fluid viscosity η^{f}. When dealing with arbitrary shapes this approximation is no longer correct. Even though F_i^{drag} is small compared to other dissipative forces, it is still used to improve the stability of the numerical integration scheme as it ensures the resistance matrix is positive definite, see further in equation of motion. [3]

Cortex viscosity

Actomyosin remodeling is modeled as an effective cortex viscosity η^{c} as per Vangheel et al. [4]. We distinguish between in-plane and out-of-plane bending viscosity. The in-plane viscous damping force between two nodes i, j with velocity $\mathbf{v}$ is given by

$$\mathbf{F}_i^{\eta^{\text{c}}} = \frac{\eta^{\text{c}} t_c}{\sqrt{3}} \left((\hat{\mathbf{n}}_{ij} \cdot (\mathbf{v}_j - \mathbf{v}_i))\hat{\mathbf{n}}_{ij} + (\hat{\mathbf{t}}_{ij} \cdot (\mathbf{v}_j - \mathbf{v}_i))\hat{\mathbf{t}}_{ij} \right),$$

where the direction of viscous forces are given by the unit vectors

$$\hat{\mathbf{n}}_{ij} = \frac{\mathbf{v}_j - \mathbf{x}_i}{\|\mathbf{v}_j - \mathbf{x}_i\|},$$

$$\hat{\mathbf{t}}_{ij} = \frac{\hat{\mathbf{n}}_A + \hat{\mathbf{n}}_B}{\|\hat{\mathbf{n}}_A + \hat{\mathbf{n}}_B\|} \times \hat{\mathbf{n}}_{ij},$$

with $\hat{\mathbf{n}}_A$, and $\hat{\mathbf{n}}_B$, the normals of the triangles making up the segment between nodes i and j. Note that we do not distinguish between bulk, extensional (η_e), and shear viscosity (η^{c}), effectively setting the Trouton ratio η_e/η^{c} to 1. In contrast, a Newtonian fluid typically has a Trouton ratio of 3.

The viscous bending moment between two adjacent triangles $A = (i, j, k), B = (i, l, j)$ with small thickness t_c arises from the rate of angle deviation $\frac{\mathrm{d}\phi}{\mathrm{d}t}$ as

$$\|\mathbf{M}_{AB}^{\eta^{\text{c}}}\| = \frac{\eta^{\text{c}} t_c^3}{3} \frac{\mathrm{d}\phi}{\mathrm{d}t}.$$

From this bending moment, we derive the lever forces on the opposing nodes,

$$\mathbf{F}_k^{\eta^{\text{c}}} = -\frac{\mathbf{M}_{AB}^{\eta^{\text{c}}} \times \hat{\mathbf{l}}_A}{l_A},$$

$$\mathbf{F}_l^{\eta^{\text{c}}} = +\frac{\mathbf{M}_{AB}^{\eta^{\text{c}}} \times \hat{\mathbf{l}}_B}{l_B},$$

with l and $\hat{\mathbf{l}}$ the length and unit vector from common axis (i, j) to lever point k or l.

For lever A, this is

$$\mathbf{l}_A = \hat{\mathbf{n}}_A \times (\mathbf{x}_j - \mathbf{x}_i),$$

$$l_A = (\mathbf{x}_k - \mathbf{x}_i) \cdot \mathbf{l}_A.$$

The forces on the common points (i, j) are weighted with the projected distance of the lever on the common axis,

$$\begin{aligned} y_{i,A} &= (\mathbf{x}_k - \mathbf{x}_i) \cdot (\mathbf{x}_j - \mathbf{x}_i), \\ y_{j,A} &= (\mathbf{x}_j - \mathbf{x}_k) \cdot (\mathbf{x}_j - \mathbf{x}_i), \\ y_{i,B} &= (\mathbf{x}_l - \mathbf{x}_i) \cdot (\mathbf{x}_j - \mathbf{x}_i), \\ y_{j,B} &= (\mathbf{x}_j - \mathbf{x}_l) \cdot (\mathbf{x}_j - \mathbf{x}_i), \end{aligned}$$

to obtain the forces on the common nodes for lever forces $F_k^{\eta^c}$ and $F_l^{\eta^c}$

$$\mathbf{F}_i^{\eta^c} = -\frac{y_{j,A}}{y_{i,A} + y_{j,A}} \mathbf{F}_k^{\eta^c} - \frac{y_{j,B}}{y_{i,B} + y_{j,B}} \mathbf{F}_l^{\eta^c},$$

$$\mathbf{F}_j^{\eta^c} = -\frac{y_{i,A}}{y_{j,A} + y_{i,A}} \mathbf{F}_k^{\eta^c} - \frac{y_{i,B}}{y_{i,B} + y_{j,B}} \mathbf{F}_l^{\eta^c},$$

that ensure force balance.

Contact forces

The contact pressure on contacting triangles AB is given by

$$P_{AB}^d(\mathbf{x}) = -\frac{k_d}{S_{AB}} \delta(\mathbf{x}), \tag{5}$$

with k_d the contact stiffness, S_{AB} the contact area, and $\delta(\mathbf{x})$ the overlapping distance.

The resulting contact forces and moment are obtained by integrating the total contact pressure over the oriented contact area $\mathbf{S}_{AB}$,

$$\mathbf{F}_{AB}^{\text{adh}} = \int \mathrm{d}\mathbf{S}_{AB}(\mathbf{x})\ P_{AB}^{\text{adh}}(\mathbf{x}). \tag{6}$$

To distribute the contact forces acting on the contact planes to the nodal contact forces $\mathbf{F}_{AB,i}^{\text{adh}}$, it is assumed that the nodal forces are collinear with the contact normal $\hat{\mathbf{n}}_{AB}$. This results in a system of linear equations per contact pair (AB)

$$\begin{aligned} \sum_{i \in A} \mathbf{F}_{AB,i}^{\text{adh}} &= -\int_{\mathbf{x} \in A \cap B} \mathrm{d}\mathbf{S}_{AB}(\mathbf{x})\ P_{AB}^{\text{adh}}(\mathbf{x}) \\ \sum_{i \in A} [(\mathbb{I} - \hat{\mathbf{n}}_{AB} \otimes \hat{\mathbf{n}}_{AB}) \cdot (\mathbf{x}_i - \mathbf{x}_{AB})] \times \mathbf{F}_{AB,i}^{\text{adh}} & \\ &= \int_{\mathbf{x} \in A \cap B} \mathrm{d}\mathbf{S}_{AB}(\mathbf{x}) \times (\mathbf{x} - \mathbf{x}_{AB})\ P_{AB}^{\text{adh}}(\mathbf{x}) \end{aligned} \tag{7}$$

The eggshell itself is assumed undeformable, meaning we don't integrate the node positions.

Wet contact friction

A viscous contact force is included to account for drag between contacting surfaces with friction constant ξ (units of Pa s/m), expressing the scaling between a dissipative traction $\mathbf{T}_c$ and the sliding velocity of two contacting surfaces A and B

$$\mathbf{T}_{AB}^{\text{fric}} = -\xi \Delta \mathbf{v}_{AB}. \tag{8}$$

In the particle-based model, the contact drag force acting on node i of triangle A of the contact pair (AB) is computed as

$$\mathbf{F}_{AB,i}^{\text{fric}} = \Gamma_{AB}^{\text{fric}} \cdot \sum_{k \in B} w_{ik}^{AB} \, (\mathbf{v}_k - \mathbf{v}_i) \tag{9}$$

determined by a friction tensor $\Gamma_{AB}^{\text{fric}}$ and weights w_{ik}^{AB} per node k of the B triangle. w_{ik}^{AB} are assumed to scale with the relative contribution of the nodal contact forces to the overall contact force thus

$$w_{ik}^{AB} = \frac{\left(\mathbf{F}_{AB,i}^{\text{adh}} + \mathbf{F}_{AB,k}^{\text{adh}}\right) \cdot \hat{\mathbf{n}}_{AB}}{6 \sum_{\forall k \in B} \mathbf{F}_{AB,k}^{\text{adh}} \cdot \hat{\mathbf{n}}_{AB}}$$

is used as an approximation. $\Gamma_{AB}^{\text{fric}}$ for a given contact area S_{AB} is estimated as

$$\Gamma_{AB}^{\text{fric}} = S_{AB} \left[\xi^{\perp} \hat{\mathbf{n}}_{AB} \otimes \hat{\mathbf{n}}_{AB} + \xi^{\parallel} \left(\mathbb{I} - \hat{\mathbf{n}}_{AB} \otimes \hat{\mathbf{n}}_{AB} \right) \right],$$

with normal and tangential friction coefficients $\xi^{\perp}$ and $\xi^{\parallel}$ respectively. Note that (9) can be equivalently formulated as

$$\mathbf{F}_{AB,i}^{\text{fric}} = \sum_{k \in B} \Gamma_{AB,ik}^{\text{fric}} \cdot (\mathbf{v}_k - \mathbf{v}_i) \,,$$

if we denote

$$\Gamma_{AB,ik}^{\text{fric}} = w_{ik}^{AB} \Gamma_{AB}^{\text{fric}}. \tag{10}$$

Actin bundle forces

Actin bundles define a chain of consecutive edges that link two foci on the mesh. Each chain acts as a contractile element with line tension and a damper that resists bending, compression, and extension. Line tension is regulated by density factor ρ as determined by

$$f(\rho) = \begin{cases} 2\,\rho, & \rho < 0.5, \\ 1, & 0.5 \le \rho \le 1, \\ \dfrac{1}{1 + (\rho - 1)^2}, & \rho > 1. \end{cases}$$

The line tension force on node i, from each connected node j, is

$$\mathbf{F}^{\mathrm{L}}_{ij} = f(\rho)\,\gamma_L\,\hat{\mathbf{n}}_{ij}, \tag{11}$$

With normal direction

$$\hat{\mathbf{n}}_{ij} = \frac{\mathbf{x}_j - \mathbf{x}_i}{\|\mathbf{x}_j - \mathbf{x}_i\|}$$

The damper part of the actin bundles is also affected by density ρ, but only in the sparse domain:

$$g(\rho) = \begin{cases} 2\,\rho, & \rho < 0.5, \\ 1, & 0.5 \le \rho. \end{cases}$$

The damping force on node i arises from its relative velocity vector $\mathbf{v}_{ij} = \mathbf{v}_j - \mathbf{v}_i$ with respect to each connected node j:

$$\begin{aligned} \mathbf{F}^{\mathrm{C}}_{ij} = {} & g(\rho)\,c_N\big(\hat{\mathbf{n}}_{ij}\cdot\mathbf{v}_{ij}\big)\hat{\mathbf{n}}_{ij} \\ & + g(\rho)\,c_T\big(\hat{\mathbf{t}}_{ij}\cdot\mathbf{v}_{ij}\big)\hat{\mathbf{t}}_{ij} \\ & + g(\rho)\,c_T\big(\hat{\mathbf{b}}_{ij}\cdot\mathbf{v}_{ij}\big)\hat{\mathbf{b}}_{ij} \end{aligned} \tag{12}$$

Where c_N is the normal damping constant, c_T the tangential damping constant, $\hat{\mathbf{n}}_{ij}$ the unit normal vector from j to i, while $\hat{\mathbf{t}}_{ij}$ and $\hat{\mathbf{b}}_{ij}$ are the two directions perpendicular to $\hat{\mathbf{n}}_{ij}$ so that the three form an orthonormal basis.

Equation of motion

In the overdamped cellular environment, inertial forces may be neglected [5]. Based on the different contributions described above, the force balance for node i gives

$$\begin{aligned} &\mathbf{F}^{\mathrm{act}}_i + \mathbf{F}^{\mathrm{cyt}}_i + \sum_j \mathbf{F}^{\mathrm{L}}_{ij} + \sum_{(AB):i\in A} \mathbf{F}^{\mathrm{adh}}_{AB,i} \\ &\qquad = \Gamma^{\mathrm{f}}_{ii}\cdot\mathbf{v}_i + \sum_j \Gamma^{\mathrm{c}}_{ij}\cdot(\mathbf{v}_i - \mathbf{v}_j) + \sum_{(AB):i\in A}\sum_{k\in B}\Gamma^{\mathrm{fric}}_{AB,ik}\cdot(\mathbf{v}_i - \mathbf{v}_k) + \sum_j \mathbf{F}^{\mathrm{C}}_{ij} \end{aligned}$$

which can be abbreviated as

$$\mathbf{F}_i = \sum_j \Gamma_{ij}\cdot\mathbf{v}_j \tag{13}$$

for a system consisting of N nodes, where

$$\Gamma_{ij} = \begin{cases} \Gamma^{\mathrm{f}}_{ii} + \sum_{k:k\neq i}\Gamma^{\mathrm{c}}_{ik} + \sum_{(AB):i\in A}\sum_{k\in B}\Gamma^{\mathrm{fric}}_{AB,ik}, & i = j, \\ -\Gamma^{\mathrm{c}}_{ij} - \sum_{(AB):i\in A, j\in B}\Gamma^{\mathrm{fric}}_{AB,ij}, & i \neq j. \end{cases}$$

The Cartesian components of the overall force vectors can be represented as a single $(3N\times 1)$ column matrix, while the friction matrices can be assembled to a single $(3N\times 3N)$ sparse symmetric positive definite friction matrix [6]. The conjugate gradient method

(CGM) is used to efficiently solve the system for the node velocities $\{\mathbf{v}_j\}$ at each iteration. Positions of the nodes are subsequently updated using a semi-implicit Euler integration scheme.

$$\mathbf{x}_i(t + \Delta t) = \mathbf{x}_i(t) + \Delta t\, \mathbf{v}_i(t + \Delta t), \tag{14}$$

where $\mathbf{v}_i(t + \Delta t)$ are the projected new velocities obtained by solving eq. 13 at time t via the CGM.

2 Supplementary Tables

Parameter	Symbol	Value	Units	Sources
Isotropic surface tension	γ	≤ 0.5	nN/µm	[7]
Local area modulus	K_A	0.001	nN/µm	helps with stability
Bulk modulus cell	K_V	1	kPa	[1]
K integral term	T_I	0.5	s	
Thickness cortex	t_c	300	µm	[8, 1]
Adhesive range	h	200	nm	
Normal Friction	$\xi^{\perp}$	1	kPa s/µm	
Tangential Friction	$\xi^{\parallel}$	0.001	kPa s/µm	
Liquid viscosity	η^{f}	150	Pa s	
Cortex viscosity	η^{c}	90	kPa s	
Diffusivity	D	2×10^{-14}	m^2/s	
Cell radius	R_0	19	µm	[1]
Simulation timestep	Δt	0.08	s	stable
Remeshing time		1	s	
Bundle line tension	γ_L	4.5	nN	
Normal damping	c_N	45	nN s/µm	10× line tension
Tangential damping	c_T	15	nN s/µm	
Minimum focus distance	d_{min}	4.5	µm	[9]
Maximum focus distance	d_{max}	9	µm	
Induction range	$r_{induction}$	4	µm	
Simulation duration		650	s	
Bundles start		20	s	
Induction start		100	s	

Table A: Simulation parameters. Three sections separate continuum properties, particle-based properties, and timings.

Parameter	Value	λ_p	v_{90}	Notes
Line tension	3	0.45	2.77	Slow, incomplete polarization, few ruffles
γ_L (nN)	4.5	0.53	4.77	Default
	6	0.54	7.62	Fast polarization, deep ruffles
Area conservation	.0005	0.54	4.82	–
K_A (nN/µm)	.001	0.53	4.77	Default
	.002	0.49	4.60	–
Shell friction	.0005	0.52	4.76	–
$\xi^{\parallel}$ (kPa s/µm)	.001	0.53	4.77	Default
	.005	0.54	4.63	–
Cortex viscosity	80	0.54	5.01	Faster polarization
η^c (kPa s)	90	0.53	4.77	Default
	100	0.53	4.49	Slower polarization
Focus distances	3–7	0.58	5.92	Faster due to more bundles
$d_{\min}$–$d_{\max}$ (µm)	4.5–9	0.53	4.77	Default
	6–11	0.52	3.80	Slower due to less bundles
Induction range	2	0.49	4.13	Slower due to weaker induction
$r_{induction}$ (µm)	4	0.53	4.77	Default
	6	0.54	5.09	Faster due to stronger induction
Normal bundle damping	30	0.52	4.77	–
c_N (nN s/µm)	45	0.53	4.77	Default
	60	0.54	4.67	–
Tangential bundle damping	5	0.53	4.53	–
c_T (nN s/µm)	15	0.53	4.77	Default
	30	0.53	4.52	–

Table B: Sensitivity analysis for physical parameters of polarization simulations. For each mutation, 10 repeats were simulated. We use two metrics to summarize the polarization flow and resulting domains. To condense the flow speeds into a single number, we use the 90th percentile (0.9 quantile) of the observed focus velocities: v_{90}. At the end of the simulation, we use the material density on the cortex mesh to quantify the final domain sizes. First, we find the plane that separates the A and P domains at the 0.1 density quantile. This yields the unit normal vector of this plane $\mathbf{n}_p$ (pointing to A) as the direction of polarization, and its signed distance to the origin d_p. We then rescale this to $\lambda_p = (d_p + L/2)/L$ with $L = 52\,\mu\text{m}$ as the typical length of the embryo. This describes a 0–1 range that covers everything from a case without polarization up to a case where the P domain covers the entire zygote. Induction ranges lower than 2 µm experienced occasional delayed or absent symmetry breaking.

References

[1] Maxim Cuvelier, Jef Vangheel, Wim Thiels, Herman Ramon, Rob Jelier, and Bart Smeets. Stability of asymmetric cell division: A deformable cell model of cytokinesis applied to c. elegans. *Biophysical Journal*, 122(10):1858–1867, May 2023. doi: 10.1016/j.bpj.2023.04.017. URL https://doi.org/10.1016/j.bpj.2023.04.017.

[2] Dmitry A. Fedosov, Bruce Caswell, and George Em Karniadakis. Systematic coarse-graining of spectrin-level red blood cell models. *Computer Methods in Applied Mechanics and Engineering*, 199(29–32):1937–1948, June 2010. ISSN 0045-7825. doi: 10.1016/j.cma.2010.02.001. URL http://dx.doi.org/10.1016/j.cma.2010.02.001.

[3] P. Van Liedekerke, B. Smeets, T. Odenthal, E. Tijskens, and H. Ramon. Solving microscopic flow problems using stokes equations in sph. *Computer Physics Communications*, 184(7):1686–1696, July 2013. ISSN 0010-4655. doi: 10.1016/j.cpc.2013.02.013. URL http://dx.doi.org/10.1016/j.cpc.2013.02.013.

[4] Jef Vangheel, Herman Ramon, and Bart Smeets. Rigidity transitions in a three-dimensional active foam model of cell monolayers with frictional contact interactions. *Physical Review Research*, 8(1), January 2026. ISSN 2643-1564. doi: 10.1103/7syt-bjgy. URL http://dx.doi.org/10.1103/7syt-bjgy.

[5] E. M. Purcell. Life at low reynolds number. *American Journal of Physics*, 45(1):3–11, January 1977. ISSN 1943-2909. doi: 10.1119/1.10903. URL http://dx.doi.org/10.1119/1.10903.

[6] Tim Odenthal, Bart Smeets, Paul Van Liedekerke, Engelbert Tijskens, Hans Van Oosterwyck, and Herman Ramon. Analysis of initial cell spreading using mechanistic contact formulations for a deformable cell model. *PLoS Computational Biology*, 9(10):e1003267, October 2013. doi: 10.1371/journal.pcbi.1003267. URL https://doi.org/10.1371/journal.pcbi.1003267.

[7] Kazunori Yamamoto, Sacha Ichbiah, Joana Pinto, Fabrice Delbary, Nate Goehring, Hervé Turlier, and Guillaume Charras. Dissecting the subcellular forces sculpting early C. elegans embryos. *bioRxiv*, March 2023. doi: 10.1101/2023.03.07.531437. URL http://dx.doi.org/10.1101/2023.03.07.531437.

[8] Bart Smeets, Maxim Cuvelier, Jiri Pešek, and Herman Ramon. The effect of cortical elasticity and active tension on cell adhesion mechanics. *Biophysical Journal*, 116(5):930–937, March 2019. doi: 10.1016/j.bpj.2019.01.015. URL https://doi.org/10.1016/j.bpj.2019.01.015.

[9] Mirjam Mayer, Martin Depken, Justin S. Bois, Frank Jülicher, and Stephan W. Grill. Anisotropies in cortical tension reveal the physical basis of polarizing cortical flows. *Nature*, 467(7315):617–621, September 2010. doi: 10.1038/nature09376. URL https://doi.org/10.1038/nature09376.
