# Supplementary material for: Nematic structures contribute to robust zygotic polarization in C. elegans
Source: PLoS Comput Biol. 2026 Sep 23;22(9):e1014792. doi: 10.1371/journal.pcbi.1014792 (PMC13630317; doi:10.1371/journal.pcbi.1014792)
Supplement: Supplementary file 2 [file pcbi.1014792.s013.pdf]

Response to Reviewers

Nematic structures contribute to robust zygotic polarization in *C. elegans*

August 17, 2026

We thank the editor for giving us the opportunity to respond and revise our manuscript. Below we address each comment in detail. Reviewer comments are in blue; adjusted text from the manuscript is in italics.

Reviewer #1

> "This study aims to elucidate the role of nematic structures—associated with discrete actin filament bundles—in shaping the mechanical properties of the actin cortex. Specifically, it seeks to clarify how nematic contributions influence the propagation of polarizing cortical flows and the formation of a spontaneous partial ingression ring (pseudocleavage) in the *C. elegans* zygote. The authors adapted an existing 3D biomechanical simulation tool to incorporate nematic structures under tension (actin filaments) connected by foci (myosin), extending prior coarse-grained models based on continuum layers. By introducing a set of hypotheses—including conditions mimicking the posterior symmetry-breaking event (via localized tension reduction) and parameterizing the system with published experimental measurements of *C. elegans* zygotic cortical mechanics—they successfully reproduced key features of in vivo cortical dynamics of the *C. elegans* zygote. The model was further validated by testing alternative parameters and hypotheses, such as a variation to reduce nematic order and counteract compression-induced alignment. The authors conclude first that alignment generates the observed tension anisotropy, second that although it does not contribute to flow dynamics or domain formation, it does promote axis convergence which aligns AP axis with the long axis of the egg, thereby confirming previous findings in the field. Developing such a simulation tool represents a significant advancement and will enable further extensions to test additional hypotheses. For this reason, I believe this tool will provide lasting value to the field beyond the scope of the current study.
>
> In my opinion here are a few points that would need additional justification:"

We thank the reviewer for her constructive comments.

Major comments

> "1- It is not entirely clear how the authors have chosen the initial number of foci and the density of bundles, their length or connectivity (branches without foci), neither how these parameters influence the subsequent simulations. One could also quantitatively detail how these evolve during the simulated polarisation process and compare it with experimental densities."

The initial number of foci was set to 40, but this value has little effect as the number quickly adjusts in response to the density rules set by the minimum and maximum inter-foci distances $d_{\min}$ and $d_{\max}$. All branches are connecting foci, and whenever new foci are created or old

ones vanish, we add or remove bundles based on a Delaunay tetrahedralization of the foci, so the number of bundles stays consistent with the density of foci.

We plotted the density of foci from simulation and from measurements. Our simulations have a focal density at the lower end of the range. This was chosen to keep the simulations lightweight and compatible with the chosen mesh triangle size, while still realistically capturing the effect of the bundles. Figure R1 is now included in the Supplementary Information (Figure S3).

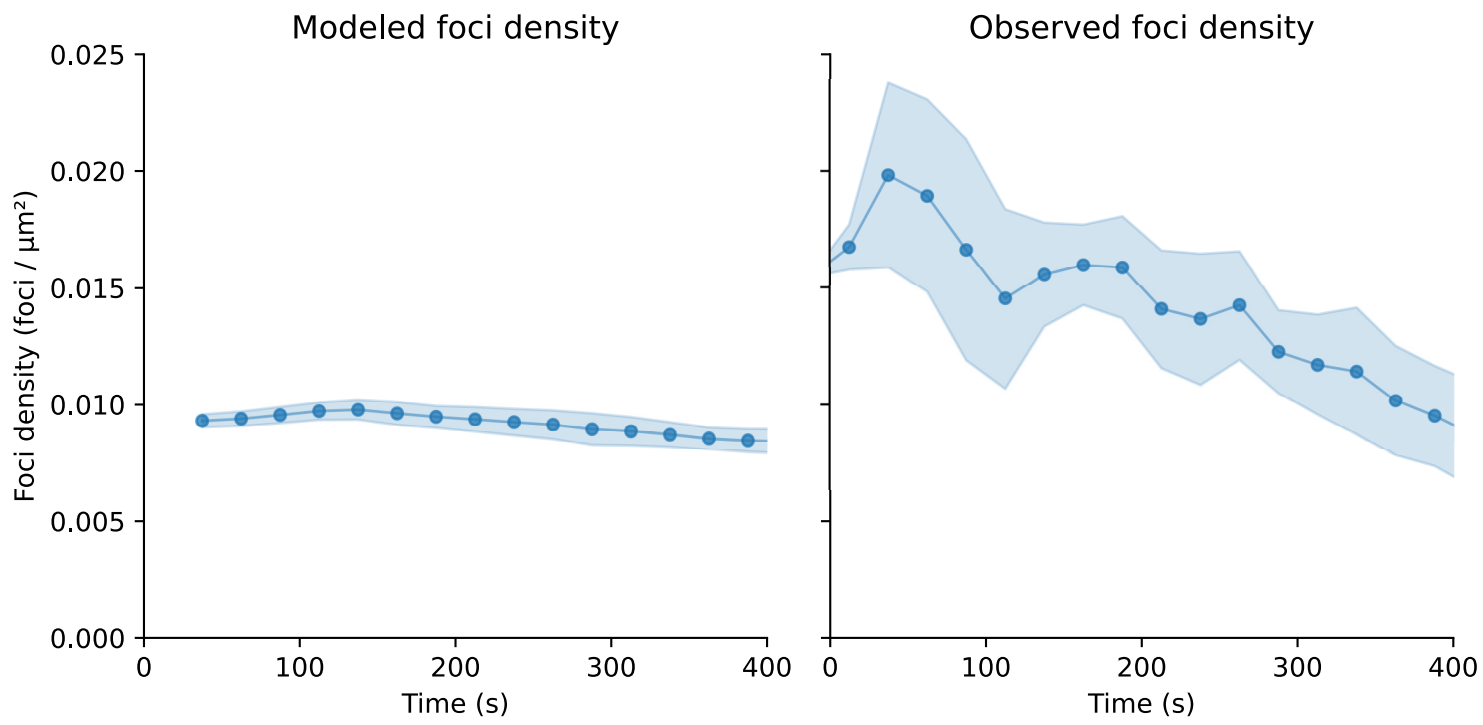

Figure R1: **Density of foci during modeled and observed polarization.** Modeled foci were excluded when $\rho \leq 1/3$, which is the case for the posterior domain where bundles are downregulated. Observed foci were counted from the same tracking used for the velocity graphs. Both figures show a downwards trend as the expanding posterior domain contains few foci. Variability is shown with standard deviations around the mean for modeled (n=20) and observed (n=6) samples.

In the sensitivity analysis (Supplementary table S2), we both increased and decreased the focus distances to investigate higher and lower focus density. This mainly resulted in a change of polarization flow velocity, as a different number of foci yields a different quantity of bundles and, with the same bundle tension, the total (anisotropic) tension changes. We have added the following clarification to the main text:

Results, Parameter tuning using flow measurements: *One example is the distance between foci ($d_{\min}$ and $d_{\max}$), which control the number of foci and bundles. This affects the flow velocity as more bundles generate more tension if the bundle tension remains unchanged.*

> "2- Rule 2 (l137) sets a decrease of bundle tension at high density. The authors proposed already several mechanisms that could justify this choice such as saturation or cadherin induced inhibition of myosin but I would also add that it could be justified more simply by a contractility induced disassembly of the acto-myosin cortex in these denser regions."

We looked into research supporting this concept and agree that it may play a role in reducing tension in the anterior cortex region. We added the following line to the justification of rule #2:

Other studies suggest that highly contractile areas, with dense NMY-2, can cause actin network disassembly due to high buckling forces (Murrell and Gardel, 2012; Wilson et al., 2010).

> "3- Precise what is the link between surface tension and bundle line tension."

In our model, the total contractility in the cortex is composed of the uniform (isotropic) surface tension (γ) acting on the mesh triangles, and the tension from actin bundles (γ_L), which is directional and can be anisotropic. These are handled separately, although cortical density

influences both. To clarify the model, we replaced 'uniform surface tension' with 'isotropic surface tension', to better distinguish it from 'bundle tension', that is directional and discrete in nature and can be anisotropic. Next, in the text we added the following lines to clarify the model.

Results, Model of the cortex: *A baseline isotropic tension is simulated over the mesh, representing contractility in the unstructured actomyosin cortex, whereas the bundle network models the tension-generating nematic structures. The two components act in parallel and additively to produce the total cortical tension.*

> "4- (l204) – The comment on the consistency of the final ratio between A and P is misleading in my opinion. Is it not directly linked to the fact that the 'flow is tuned to arrest' at this ratio 1:1 as stated in l86. Can the authors clarify these points, what is exactly imposed or left free in the simulation? Note that this is also relevant for the section in the discussion (l320)."

We think the text from the section "Model of the cortex" was confusing on the topic of tuning. We refer to the section 'Parameter tuning using flow measurement' for the more complete description, specifically:

We fine-tuned model parameters by fitting simulated flow to the experimental flow profiles. Bundle line tension $\gamma_L = 4.5\,\mathrm{nN}$ was chosen to halt polarization near the embryo midpoint. Significantly lower values result in incomplete polarization, while higher values drive the domain boundary too far anteriorly (Supplementary Table S2). Polarization speed is primarily controlled by cortical viscosity, which we chose at $\eta^c = 90\,\mathrm{kPas}$ to match observed flow speeds and duration (Figure 2B).

For clarity, when a simulation runs, there is no correction for flow or domain ratio; simulation behavior follows from the initial parameters. The discussion on the robustness of model outcomes referenced here (l204) relates to observing similar outcomes in the size ratio, despite randomness in bundle network structure or modifications of many parameters, which shows that we are in a stable (robust) regime. We highlighted the effect of the bundle line tension in our model, as it directly influences the force balance that controls where the domain boundary falls.

To improve the text, we removed the following lines from the section 'Model of the cortex', as these points are introduced clearly just a bit later in the text.

~~*Simulations start with a uniform distribution of cortical material. Symmetry is broken at a defined location by a local drop in tension, after which cortical flow arises spontaneously with a velocity profile comparable to what is observed in vivo. The flow is tuned to arrest when the domain boundary reaches the middle of the embryo, and a stable configuration of anterior and posterior domains is achieved with a size ratio of approximately 1:1.*~~

> "5- The formation of a pseudocleavage ingression in vivo is consisting of deeper invagination than those observed in the simulations. In vivo the membrane is zippering tightly as the ingression progresses quite deeply towards the cell centre. Maybe add a section to discuss this difference in the discussion. Can this be reproduced in the simulations? How does this contribute to force balance as flow cease?"

Mesh discretization limits how closely bundles can stack, which most affects the deepest invaginations, where a tensile strand would otherwise 'fall into' the groove. The current model lets bundles merge into one, but we don't add their tension together. Merging bundles and summing their tension was attempted but caused unstable simulations. A dynamic mesh size, allowing bundles to stack more closely without fully collapsing into each other, could resolve this, but would add considerable complexity to the model. In our model, invaginations don't

affect the force balance that drives cortical flow, since the forces point inwards and cancel out with surface tension. We added a comment to the discussion that addresses this limitation.

Discussion: *The pseudocleavage furrow is more pronounced in vivo than in our simulations. In our current model, mesh resolution sets a limit on how tightly bundles can converge, which likely underestimates invagination depth. A dynamic mesh size would allow bundles to stack more closely, producing deeper invaginations.*

> "6- (l303) The authors discuss the persistence of alignment by flow as actin filaments turnover occurs at faster time scales. However, they do not mention that it could be sustained longer than the individual turnover of actin filaments due to assembly with preferred orientation along the pre-existing orientation, as published notably in the context of cytokinesis by Li and Munro, 2021, Developmental Cell (ref should be included in my opinion)."

We thank the reviewer for pointing out this mechanism and agree that it may play a role in sustaining fiber alignment despite rapid turnover. This is now included in the discussion.

First, individual actin filaments turn over rapidly (10–30 s) (Mayer et al., 2010; Saha et al., 2016; Robin et al., 2014), which limits persistent alignment by flow. Instead, the cortex contains pre-existing nematic structures in the form of actin bundles with associated myosin foci that persist for minutes (Munro et al., 2004) and are present before polarization. Actin filament alignment may outlive filament lifespans through templated assembly along pre-existing orientations, as shown for the contractile ring during cytokinesis (Li and Munro, 2021). Our framework abstracts this structural memory into macroscopic actin bundles.

> "7- The authors did not state whether the computational code is or will be fully available at publication."

The code is shared publicly through bitbucket and zenodo, and the links are given in the Availability section, after the abstract.

Minor comments

> "What is the rational for the choice of manually tracking foci when automated methods are performant?"

The lab had recently developed new tracking software, and the process of tracking these 6 polarization videos was relatively quick.

> "Figure 3E: add a schematic next to the colour-scale to illustrate the signification of Q (described in main text l234)."

We have added a schematic next to the colour scale in Figure 3E to illustrate the sign of nematic order Q.

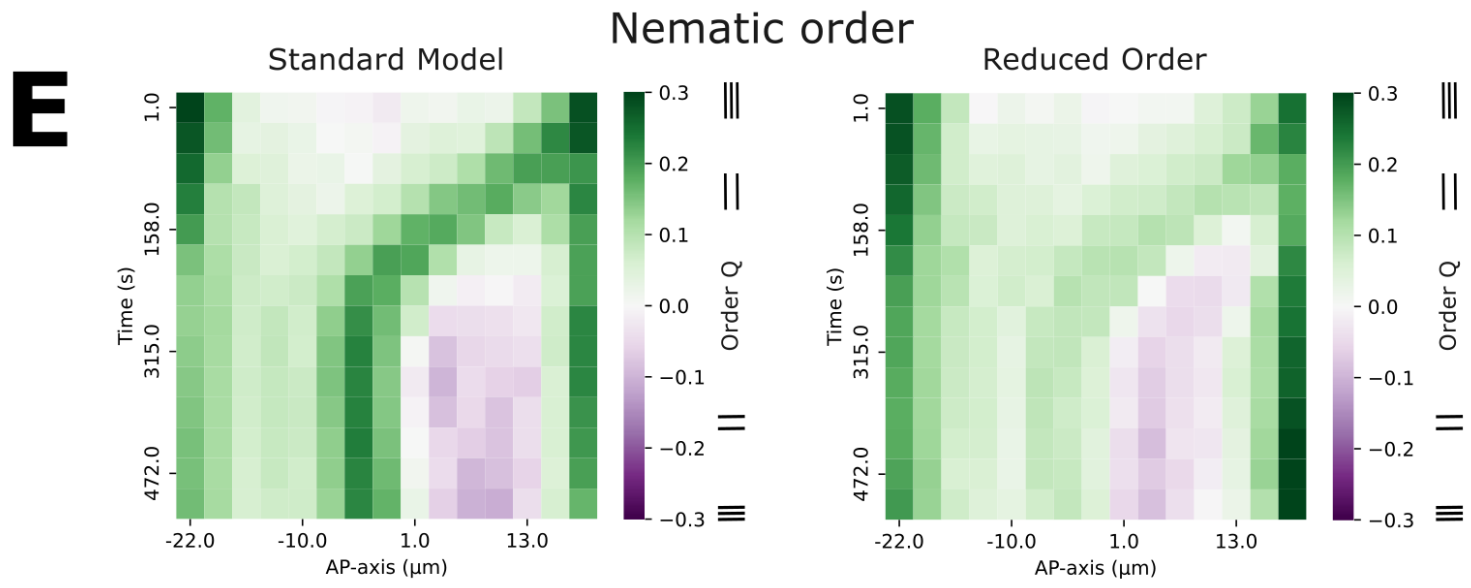

Figure R2: Nematic order Q in Figure 3E.

Reviewer #2

> "In the work by M. Vanslambrouck et al., the authors develop a mesoscopic model to describe *C. elegans* zygote polarization and compare its predictions with experimental spatiotemporal maps of cortical flow. The authors first provide a clear description of the state of the art and explain the main differences between the present theoretical model and previous studies. They then introduce their model in a descriptive manner. Next, the authors discuss two phenomenological relationships between the elastic parameters and the local actin density and present the experimental evidence supporting them. The authors then fit the model parameters to reproduce experimentally measured spatiotemporal patterns of cortical flow, achieving satisfactory agreement with the data. In a supplementary table, they briefly describe the outcomes observed when selected parameters are increased or decreased a few fold. Using their model, the authors further investigate the role of nematic alignment of actin filaments in *C. elegans* zygote polarization. They find that nematic alignment is not essential for reproducing cortical flow patterns but is required to explain axis convergence following lateral symmetry breaking. However, these theoretical predictions are not experimentally tested.
>
> The study is a solid theoretical contribution that investigates how nematic alignment influences processes involved in *C. elegans* zygote polarization. The authors compare the predictions of their theoretical model with experimental data and obtain satisfactory agreement. They also provide extensive supplementary information containing additional analyses that will be valuable to readers. Overall, this is a remarkable contribution to the field of biophysics, and I recommend publication of the manuscript. There are, however, a few minor issues that the authors may wish to address before publication."

We thank the reviewer for their constructive comments.

Minor comments

> "1. In lines 31–32, the authors state that the flow occurs over a length scale of approximately 14 micrometers and describe this scale as "large." It would be helpful to clarify what this scale is being compared to."

A hydrodynamic length scale of 14µm is large compared to the *C. elegans* embryo, which measures 55µm along its long axis. It implies that force transduction can happen over a distance of almost a third of the embryo's length; i.e., the cortex is highly viscous relative to the friction it experiences with its surroundings.

> “2. In the section ‘Model of the Cortex’, the authors adopt a descriptive rather than a mathematical approach to introduce their model, with the full details provided in the Supplementary Material. While I understand the rationale behind this choice, I found it difficult in several instances to connect the parameter definitions given in the main text with the corresponding equations in the Supplementary Material. For example, not all parameters listed in Table S1 are introduced in the main text. Moreover, the main text refers to a single viscosity, whereas Table S1 includes two viscosities, one associated with the actin cortex and another with the surrounding fluid. In the Supplementary Material, the cortex viscosity is denoted by (η^c, see Eq. below 4), while in Table S1 it appears as (η). It is unclear whether these symbols refer to the same quantity. This is only one example, and the list is not exhaustive. The manuscript would benefit from a more direct correspondence between the model description presented in the section “Model of the Cortex” and the mathematical formulation provided in the Supplementary Material.”

We thank the reviewer for pointing out these inconsistencies and added several references to the parameter definitions. Next, cortex viscosity was accidentally termed by both η and η^c, now we adopted η^c everywhere. One instance of bulk modulus K_V was displayed as K in the supplementary text, and is now corrected.

We chose to keep some parameters out of the Results section, only referencing them in Materials and Methods or the SI, as they were not directly relevant for the presentation of the results.

> “3. In lines 160–162, the authors present experimental measurements of cortical flow. However, the reported mean values are not accompanied by error bars, and the number of experiments or biological replicates is not specified. As a result, it is difficult for a non-expert reader to assess the significance of the reported differences, such as those between 4.3 micrometer/min and 9.6 micrometer/min. This comment also applies to other experimental values reported throughout the manuscript.”

We have now added clarification about the 6 wild type repeats and added standard deviations (n=6) to the velocity metrics.

Myosin foci were manually tracked over time to obtain high-resolution flow profiles (6 repeats, see Materials and Methods). A smooth spatiotemporal flow profile fit to data from all embryos analyzed is shown in Fig. 2A (see Materials and Methods). Shortly after symmetry breaking, the posterior-to-anterior flow arises with a steep speed gradient, reaching average flow rates of $(4.21 \pm 0.56)\,\mu\text{m/min}$ *from* $150\,\text{s}$ *to* $200\,\text{s}$ *(across the AP axis) while peaking at* $(9.39 \pm 1.14)\,\mu\text{m/min}$ *near the posterior pole before declining.*

Equally so for the simulated flow, but then with n=20:

Unless noted otherwise, polarization simulations were repeated 20 times with randomized bundle networks. To quantify the agreement between simulations and experiments, we compared key metrics of cortical flow dynamics. The simulations reproduced the rapid onset of cortical flow and the AP speed gradient observed experimentally, with peak velocities of $(7.39 \pm 0.91)\,\mu\text{m/min}$ *occurring around* $160\,\text{s}$ *after flow initiation in the posterior region.*

Since the flow speed kymographs (Figure 2A-B) do not show variability, we also provide velocity graphs in Figure 2C-D with 95% confidence intervals for several periods during polarization.

> “4. In Table S2, the authors provide a qualitative description of how the model behaves when certain parameters are varied by a factor of two. It would be preferable to complement these statements with corresponding figures in the Supplementary Information to support and illustrate these effects for at least some of the parameters.”

We added a new supplementary figure (Figure S4) to visualize the parameters that had an observable effect on the polarizing flow.

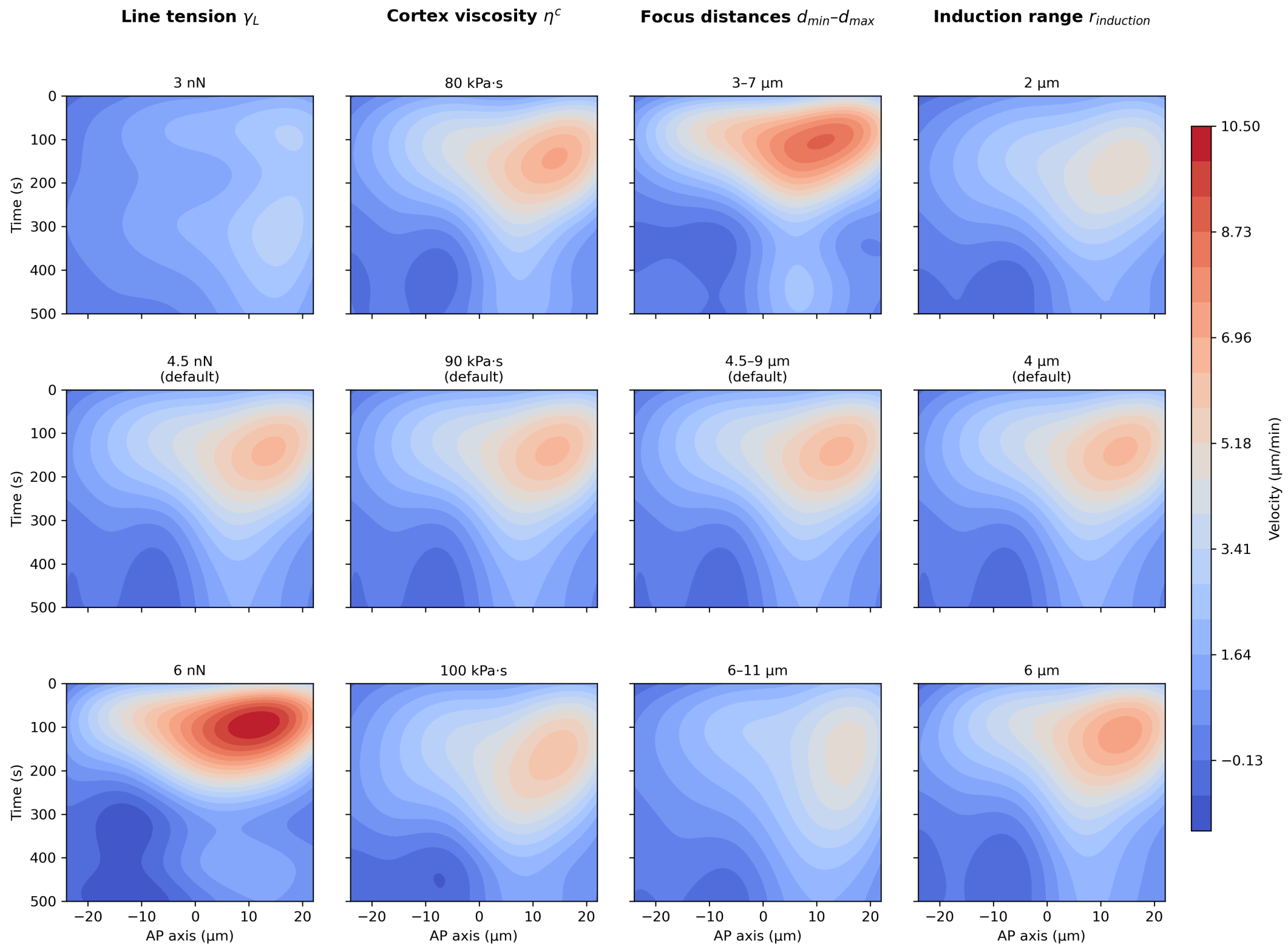

Figure R3: **Simulated flow speed kymographs visualizing the sensitivity analysis of Table S2.** Each column shows the flow speed profile for an influential parameter varied around its default value. Increasing line tension or induction range advances and intensifies the flow peak, whereas increasing cortex viscosity or focus distances (fewer bundles) delays and weakens it. These trends match the v_{90} values reported in Table S2.

> "5. In Fig. 2C and 2D, it is unclear what the solid lines represent. Are they theoretical fits or another type of fit? This information should be included in the figure caption. In addition, the colorbar in panels A and B should be made consistent to facilitate comparison between the two. The authors may also consider plotting the normalized magnitude of the cortical speed instead of the absolute magnitude. A similar comment applies to Fig. 3F."

The solid lines are smoothing splines, fit to the binned means along the AP axis. This information is now included in the figure caption. Next, we equalized the colorbars in figure 2A and 2B, and did the same within figure 3F.

> "6. In the section on axis convergence, it would be useful to compare the theoretical predictions with experimental data. The authors can discuss how the values of the AP angular deviation shown in Fig. 4D compare with those reported in previous experimental studies."

Bhatnagar et al. (2023) report that AP axis convergence in unperturbed embryos reliably reaches near-complete alignment (average below 5°) within about 7 minutes. We report a

small final axis error of 14°, which hence contains a residual alignment error not observed in actual embryos. There are several potential explanations, with a known limitation of the DCM as the likely main culprit: limited long-distance force transmission by overestimated restrictive forces. Other potential explanations are that the actual embryos start with a milder initial misalignment than the reported simulation output, and that we do not capture the cytoplasmic flow, which Bhatnagar et al. (2023) reported as having a modest additional effect.

Several extensions could improve the model's realism. The axis convergence simulations reveal a small residual error and slower dynamics. Our diagnosis is that the DCM provides an incomplete capture of long-distance transmission of viscous forces, which may be refined in future versions. Further, we also did not capture the pronuclei or cytoplasmic flow, which may provide a small additive effect (Bhatnagar et al., 2023).

> "7. In Fig. S1, the authors may consider adding a panel showing the relative error map."

We considered a relative error map. However, direct normalization by observed velocity is ill-behaved since velocities approach and cross zero. Alternatively, dividing by the maximum observed velocity is an option, but the resulting map is proportional to the original plot and adds no additional value.

References

A. Bhatnagar, M. Nestler, P. Gross, M. Kramar, M. Leaver, A. Voigt, and S. W. Grill. Axis convergence in c. elegans embryos. *Current Biology*, 33(23):5096–5108.e15, Dec. 2023. ISSN 0960-9822. doi: 10.1016/j.cub.2023.10.050. URL http://dx.doi.org/10.1016/j.cub.2023.10.050.

Y. Li and E. Munro. Filament-guided filament assembly provides structural memory of filament alignment during cytokinesis. *Developmental Cell*, 56(17):2486–2500.e6, 2021. ISSN 1534-5807. doi: 10.1016/j.devcel.2021.08.009. URL http://dx.doi.org/10.1016/j.devcel.2021.08.009.

M. Mayer, M. Depken, J. S. Bois, F. Jülicher, and S. W. Grill. Anisotropies in cortical tension reveal the physical basis of polarizing cortical flows. *Nature*, 467(7315):617–621, Sept. 2010. doi: 10.1038/nature09376. URL https://doi.org/10.1038/nature09376.

E. Munro, J. Nance, and J. R. Priess. Cortical flows powered by asymmetrical contraction transport par proteins to establish and maintain anterior-posterior polarity in the early c. elegans embryo. *Developmental Cell*, 7(3):413–424, Sept. 2004. ISSN 1534-5807. doi: 10.1016/j.devcel.2004.08.001. URL http://dx.doi.org/10.1016/j.devcel.2004.08.001.

M. P. Murrell and M. L. Gardel. F-actin buckling coordinates contractility and severing in a biomimetic actomyosin cortex. *Proceedings of the National Academy of Sciences*, 109 (51):20820–20825, Dec. 2012. ISSN 1091-6490. doi: 10.1073/pnas.1214753109. URL http://dx.doi.org/10.1073/pnas.1214753109.

F. B. Robin, W. M. McFadden, B. Yao, and E. M. Munro. Single-molecule analysis of cell surface dynamics in caenorhabditis elegans embryos. *Nature Methods*, 11(6):677–682, Apr. 2014. ISSN 1548-7105. doi: 10.1038/nmeth.2928. URL http://dx.doi.org/10.1038/nmeth.2928.

A. Saha, M. Nishikawa, M. Behrndt, C.-P. Heisenberg, F. Jülicher, and S. W. Grill. Determining physical properties of the cell cortex. *Biophysical Journal*, 110(6):1421–1429, Mar. 2016. doi: 10.1016/j.bpj.2016.02.013. URL https://doi.org/10.1016/j.bpj.2016.02.013.

C. A. Wilson, M. A. Tsuchida, G. M. Allen, E. L. Barnhart, K. T. Applegate, P. T. Yam, L. Ji, K. Keren, G. Danuser, and J. A. Theriot. Myosin ii contributes to cell-scale actin network

treadmilling through network disassembly. *Nature*, 465(7296):373–377, May 2010. ISSN 1476-4687. doi: 10.1038/nature08994. URL http://dx.doi.org/10.1038/nature08994.
