# Supplementary material for: Nematic structures contribute to robust zygotic polarization in C. elegans
Source: PLoS Comput Biol. 2026 Sep 23;22(9):e1014792. doi: 10.1371/journal.pcbi.1014792 (PMC13630317; doi:10.1371/journal.pcbi.1014792)
Supplement: Peer review history

# Peer review: Decision Letter 0

**DOI:** 10.1371/journal.pcbi.1014792.r001

**Authors:** Dimitrios Vavylonis, Calina Copos

17 Jun 2026

PCOMPBIOL-D-26-00903

Nematic structures contribute to robust zygotic polarization in C. elegans

PLOS Computational Biology

Dear Dr. Jelier,

Thank you for submitting your manuscript to PLOS Computational Biology. After careful consideration, we feel that it has merit but does not fully meet PLOS Computational Biology's publication criteria as it currently stands. Therefore, we invite you to submit a revised version of the manuscript that addresses the points raised during the review process.

Please submit your revised manuscript by Aug 17 2026 11:59PM. If you will need more time than this to complete your revisions, please reply to this message or contact the journal office at ploscompbiol@plos.org. When you're ready to submit your revision, log on to https://www.editorialmanager.com/pcompbiol/ and select the 'Submissions Needing Revision' folder to locate your manuscript file.

Please include the following items when submitting your revised manuscript:

* A letter that responds to each point raised by the editor and reviewer(s). You should upload this letter as a separate file labeled 'Response to Reviewers'. This file does not need to include responses to formatting updates and technical items listed in the 'Journal Requirements' section below.

* A marked-up copy of your manuscript that highlights changes made to the original version. You should upload this as a separate file labeled 'Revised Manuscript with Track Changes'.

* An unmarked version of your revised paper without tracked changes. You should upload this as a separate file labeled 'Manuscript'.

If you would like to make changes to your financial disclosure, competing interests statement, or data availability statement, please make these updates within the submission form at the time of resubmission. Guidelines for resubmitting your figure files are available below the reviewer comments at the end of this letter.

As the corresponding author, your ORCID iD is verified in the submission system and will appear in the published article. PLOS supports the use of ORCID, and we encourage all coauthors to register for an ORCID iD and use it as well. Please encourage your coauthors to verify their ORCID iD within the submission system before final acceptance, as unverified ORCID iDs will not appear in the published article. Only the individual author can complete the verification step; PLOS staff cannot verify ORCID iDs on behalf of authors.

We look forward to receiving your revised manuscript.

Kind regards,

Calina Copos, Ph.D.

Academic Editor

PLOS Computational Biology

Dimitrios Vavylonis

Section Editor

PLOS Computational Biology

**Journal Requirements:**

If the reviewer comments include a recommendation to cite specific previously published works, please review and evaluate these publications to determine whether they are relevant and should be cited. There is no requirement to cite these works unless the editor has indicated otherwise.

1) We ask that a manuscript source file is provided at Revision. Please upload your manuscript file as a .doc, .docx, .rtf or .tex. If you are providing a .tex file, please upload it under the item type u2018LaTeX Source Fileu2019 and leave your .pdf version as the item type u2018Manuscriptu2019.

2) Please provide an Author Summary. This should appear in your manuscript between the Abstract (if applicable) and the Introduction, and should be 150-200 words long. The aim should be to make your findings accessible to a wide audience that includes both scientists and non-scientists. Sample summaries can be found on our website under Submission Guidelines:

https://journals.plos.org/ploscompbiol/s/submission-guidelines#loc-parts-of-a-submission

3) Please upload all main figures as separate Figure files in .tif or .eps format. For more information about how to convert and format your figure files please see our guidelines:

https://journals.plos.org/ploscompbiol/s/figures

4) Please upload a copy of Figures 1 A-C, 2A-F, 3A-F, 4A-D, and 5 which you refer to in your text on pages 2, 5, 7, 8, and 14. Or, if the figure is no longer to be included as part of the submission please remove all reference to it within the text.

5) We have noticed that you have uploaded Supporting Information files, but you have not included a list of legends. Please add a full list of legends for your Supporting Information files after the references list.

6) Please amend your detailed Financial Disclosure statement. This is published with the article. It must therefore be completed in full sentences and contain the exact wording you wish to be published.

1) State the initials, alongside each funding source, of each author to receive each grant. For example: "This work was supported by the National Institutes of Health (####### to AM; ###### to CJ) and the National Science Foundation (###### to AM)."

2) State what role the funders took in the study. If the funders had no role in your study, please state: "The funders had no role in study design, data collection and analysis, decision to publish, or preparation of the manuscript."

3) If any authors received a salary from any of your funders, please state which authors and which funders..

If you did not receive any funding for this study, please simply state: u201cThe authors received no specific funding for this work.u201d

7)We have amended your Competing Interest statement to comply with journal style. We kindly ask that you double check the statement and let us know if anything is incorrect.

**Reviewers' comments:**

Reviewer's Responses to Questions

**Comments to the Authors:**

**Please note here if the review is uploaded as an attachment.**

Reviewer #1: This study aims to elucidate the role of nematic structures—associated with discrete actin filament bundles—in shaping the mechanical properties of the actin cortex. Specifically, it seeks to clarify how nematic contributions influence the propagation of polarizing cortical flows and the formation of a spontaneous partial ingression ring (pseudocleavage) in the C. elegans zygote.

The authors adapted an existing 3D biomechanical simulation tool to incorporate nematic structures under tension (actin filaments) connected by foci (myosin), extending prior coarse-grained models based on continuum layers. By introducing a set of hypotheses—including conditions mimicking the posterior symmetry-breaking event (via localized tension reduction) and parameterizing the system with published experimental measurements of C. elegans zygotic cortical mechanics—they successfully reproduced key features of in vivo cortical dynamics of the C. elegans zygote. The model was further validated by testing alternative parameters and hypotheses, such as a variation to reduce nematic order and counteract compression-induced alignment.

The authors conclude first that alignment generates the observed tension anisotropy, second that although it does not contribute to flow dynamics or domain formation, it does promote axis convergence which aligns AP axis with the long axis of the egg, thereby confirming previous findings in the field.

Developing such a simulation tool represents a significant advancement and will enable further extensions to test additional hypotheses. For this reason, I believe this tool will provide lasting value to the field beyond the scope of the current study.

In my opinion here are a few points that would need additional justification:

1- It is not entirely clear how the authors have chosen the initial number of foci and the density of bundles, their length or connectivity (branches without foci), neither how these parameters influence the subsequent simulations. One could also quantitatively detail how these evolve during the simulated polarisation process and compare it with experimental densities.

2- Rule 2 (l137) sets a decrease of bundle tension at high density. The authors proposed already several mechanisms that could justify this choice such as saturation or cadherin induced inhibition of myosin but I would also add that it could be justified more simply by a contractility induced disassembly of the acto-myosin cortex in these denser regions.

3- Precise what is the link between surface tension and bundle line tension.

4- (l204) – The comment on the consistency of the final ratio between A and P is misleading in my opinion. Is it not directly linked to the fact that the “flow is tuned to arrest” at this ratio 1:1 as stated in l86. Can the authors clarify these points, what is exactly imposed or left free in the simulation?

Note that this is also relevant for the section in the discussion (l320).

5- The formation of a pseudocleavage ingression in vivo is consisting of deeper invagination than those observed in the simulations. In vivo the membrane is zippering tightly as the ingression progresses quite deeply towards the cell centre. Maybe add a section to discuss this difference in the discussion. Can this be reproduced in the simulations? How does this contribute to force balance as flow cease?

6- (l303) The authors discuss the persistence of alignment by flow as actin filaments turnover occurs at faster time scales. However, they do not mention that it could be sustained longer than the individual turnover of actin filaments due to assembly with preferred orientation along the pre-existing orientation, as published notably in the context of cytokinesis by Li and Munro, 2021, Developmental Cell (ref should be included in my opinion).

7- The authors did not state whether the computational code is or will be fully available at publication.

Minor comments:

-What is the rational for the choice of manually tracking foci when automated methods are performant?

-Figure 3E: add a schematic next to the colour-scale to illustrate the signification of Q (described in main text l234).

Reviewer #2: In the work by M. Vanslambrouck et al., the authors develop a mesoscopic model to describe C. elegans zygote polarization and compare its predictions with experimental spatiotemporal maps of cortical flow. The authors first provide a clear description of the state of the art and explain the main differences between the present theoretical model and previous studies. They then introduce their model in a descriptive manner. Next, the authors discuss two phenomenological relationships between the elastic parameters and the local actin density and present the experimental evidence supporting them. The authors then fit the model parameters to reproduce experimentally measured spatiotemporal patterns of cortical flow, achieving satisfactory agreement with the data. In a supplementary table, they briefly describe the outcomes observed when selected parameters are increased or decreased a few fold. Using their model, the authors further investigate the role of nematic alignment of actin filaments in C. elegans zygote polarization. They find that nematic alignment is not essential for reproducing cortical flow patterns but is required to explain axis convergence following lateral symmetry breaking. However, these theoretical predictions are not experimentally tested.

The study is a solid theoretical contribution that investigates how nematic alignment influences processes involved in C. elegans zygote polarization. The authors compare the predictions of their theoretical model with experimental data and obtain satisfactory agreement. They also provide extensive supplementary information containing additional analyses that will be valuable to readers. Overall, this is a remarkable contribution to the field of biophysics, and I recommend publication of the manuscript. There are, however, a few minor issues that the authors may wish to address before publication.

Minor comments:

1. In lines 31–32, the authors state that the flow occurs over a length scale of approximately 14 micrometers and describe this scale as "large." It would be helpful to clarify what this scale is being compared to.

2.- In the section “Model of the Cortex”, the authors adopt a descriptive rather than a mathematical approach to introduce their model, with the full details provided in the Supplementary Material. While I understand the rationale behind this choice, I found it difficult in several instances to connect the parameter definitions given in the main text with the corresponding equations in the Supplementary Material. For example, not all parameters listed in Table S1 are introduced in the main text. Moreover, the main text refers to a single viscosity, whereas Table S1 includes two viscosities, one associated with the actin cortex and another with the surrounding fluid. In the Supplementary Material, the cortex viscosity is denoted by (\eta^c, see Eq. below 4), while in Table S1 it appears as (\eta). It is unclear whether these symbols refer to the same quantity. This is only one example, and the list is not exhaustive. The manuscript would benefit from a more direct correspondence between the model description presented in the section “Model of the Cortex” and the mathematical formulation provided in the Supplementary Material.

3. In lines 160–162, the authors present experimental measurements of cortical flow. However, the reported mean values are not accompanied by error bars, and the number of experiments or biological replicates is not specified. As a result, it is difficult for a non-expert reader to assess the significance of the reported differences, such as those between 4.3 micrometer/min and 9.6 micrometer/min. This comment also applies to other experimental values reported throughout the manuscript.

4. In Table S2, the authors provide a qualitative description of how the model behaves when certain parameters are varied by a factor of two. It would be preferable to complement these statements with corresponding figures in the Supplementary Information to support and illustrate these effects for at least some of the parameters.

5. In Fig. 2C and 2D, it is unclear what the solid lines represent. Are they theoretical fits or another type of fit? This information should be included in the figure caption. In addition, the colorbar in panels A and B should be made consistent to facilitate comparison between the two. The authors may also consider plotting the normalized magnitude of the cortical speed instead of the absolute magnitude. A similar comment applies to Fig. 3F.

6. In the section on axis convergence, it would be useful to compare the theoretical predictions with experimental data. The authors can discuss how the values of the AP angular deviation shown in Fig. 4D compare with those reported in previous experimental studies.

7. In Fig. S1, the authors may consider adding a panel showing the relative error map.

**********

**Have the authors made all data and (if applicable) computational code underlying the findings in their manuscript fully available?**

The PLOS Data policy requires authors to make all data and code underlying the findings described in their manuscript fully available without restriction, with rare exception (please refer to the Data Availability Statement in the manuscript PDF file). The data and code should be provided as part of the manuscript or its supporting information, or deposited to a public repository. For example, in addition to summary statistics, the data points behind means, medians and variance measures should be available. If there are restrictions on publicly sharing data or code —e.g. participant privacy or use of data from a third party—those must be specified.

Reviewer #1: **No:**  please make sure to correct this point in the revised version

Reviewer #2: Yes

**********

PLOS authors have the option to publish the peer review history of their article (what does this mean?). If published, this will include your full peer review and any attached files.

If you choose “no”, your identity will remain anonymous but your review may still be made public.

**Do you want your identity to be public for this peer review?**  For information about this choice, including consent withdrawal, please see our Privacy Policy.

Reviewer #1: **Yes:**  Anne-Cécile Reymann

Reviewer #2: No

[NOTE: If reviewer comments were submitted as an attachment file, they will be attached to this email and accessible via the submission site. Please log into your account, locate the manuscript record, and check for the action link "View Attachments". If this link does not appear, there are no attachment files.]

**Figure resubmission:**

**Reproducibility:**

To enhance the reproducibility of your results, we recommend that authors of applicable studies deposit laboratory protocols in protocols.io, where a protocol can be assigned its own identifier (DOI) such that it can be cited independently in the future. Additionally, PLOS ONE offers an option to publish peer-reviewed clinical study protocols. Read more information on sharing protocols at https://plos.org/protocols?utm_medium=editorial-email&utm_source=authorletters&utm_campaign=protocols

# Author comment: Author response to Decision Letter 1

**DOI:** 10.1371/journal.pcbi.1014792.r002

17 Aug 2026

Attachment Submitted filename: reply.pdf

# Peer review: Decision Letter 1

**DOI:** 10.1371/journal.pcbi.1014792.r003

**Authors:** Dimitrios Vavylonis, Calina Copos

2 Sep 2026

Dear Dr Jelier,

We are pleased to inform you that your manuscript 'Nematic structures contribute to robust zygotic polarization in C. elegans' has been provisionally accepted for publication in PLOS Computational Biology.

Before your manuscript can be formally accepted you will need to complete some formatting changes, which you will receive in a follow up email. A member of our team will be in touch with a set of requests.

Please note that your manuscript will not be scheduled for publication until you have made the required changes, so a swift response is appreciated.

IMPORTANT: The editorial review process is now complete. PLOS will only permit corrections to spelling, formatting or significant scientific errors from this point onwards. Requests for major changes, or any which affect the scientific understanding of your work, will cause delays to the publication date of your manuscript.

Should you, your institution's press office or the journal office choose to press release your paper, you will automatically be opted out of early publication. We ask that you notify us now if you or your institution is planning to press release the article. All press must be co-ordinated with PLOS.

Thank you again for supporting Open Access publishing; we are looking forward to publishing your work in PLOS Computational Biology.

Best regards,

Calina Copos, Ph.D.

Academic Editor

PLOS Computational Biology

Dimitrios Vavylonis

Section Editor

PLOS Computational Biology

***********************************************************

Reviewer's Responses to Questions

**Comments to the Authors:**

**Please note here if the review is uploaded as an attachment.**

Reviewer #1: The authors have addressed all the points raised during the review process and have made the necessary corrections and improvements to the text and figures accordingly.

Reviewer #2: The authors have addressed my minor concerns, and I recommend publication of the present manuscript in PLOS Computational Biology.

Minor point 1 asked for some clarification of the interpretation of a length scale; the authors have modified the text to address this comment. Minor point 2 asked to homogenise the notation between the main text and the supplementary material; the authors have introduced modifications to both texts to address this comment. Minor point 3 asked to add error bars and the number of replicates to the experimental and simulation averaged values; the authors have made modifications to the text to address this comment. Major point 4 asked to add a supplementary figure to further support the qualitative description of states in Table S2; the authors added this new figure and modified the text. Minor point 5 asked for clarification on Fig. 2; the authors have included the information in the caption. Minor point 6 suggested comparing experimental results to simulations; the authors added a new paragraph in the text discussing potential limitations of their model. Minor point 7 suggested adding a panel of the relative error of velocity measures; the authors preferred not to include such a panel and provided a justification, which sounds reasonable to me. No further minor comments were created by the modification of the reviewed manuscript.

**********

**Have the authors made all data and (if applicable) computational code underlying the findings in their manuscript fully available?**

The PLOS Data policy requires authors to make all data and code underlying the findings described in their manuscript fully available without restriction, with rare exception (please refer to the Data Availability Statement in the manuscript PDF file). The data and code should be provided as part of the manuscript or its supporting information, or deposited to a public repository. For example, in addition to summary statistics, the data points behind means, medians and variance measures should be available. If there are restrictions on publicly sharing data or code —e.g. participant privacy or use of data from a third party—those must be specified.

Reviewer #1: Yes

Reviewer #2: Yes

**********

PLOS authors have the option to publish the peer review history of their article (what does this mean?). If published, this will include your full peer review and any attached files.

If you choose “no”, your identity will remain anonymous but your review may still be made public.

**Do you want your identity to be public for this peer review?**  For information about this choice, including consent withdrawal, please see our Privacy Policy.

Reviewer #1: **Yes:**  Anne-Cécile Reymann

Reviewer #2: No

# Editor report: Acceptance letter

**DOI:** 10.1371/journal.pcbi.1014792.r004

**Authors:** Dimitrios Vavylonis, Calina Copos

PCOMPBIOL-D-26-00903R1

Nematic structures contribute to robust zygotic polarization in C. elegans

Dear Dr Jelier,

I am pleased to inform you that your manuscript has been formally accepted for publication in PLOS Computational Biology. Your manuscript is now with our production department and you will be notified of the publication date in due course.

The corresponding author will soon be receiving a typeset proof for review, to ensure errors have not been introduced during production. Please review the PDF proof of your manuscript carefully, as this is the last chance to correct any errors. Please note that major changes, or those which affect the scientific understanding of the work, will likely cause delays to the publication date of your manuscript.

Soon after your final files are uploaded, unless you have opted out, the early version of your manuscript will be published online. The date of the early version will be your article's publication date. The final article will be published to the same URL, and all versions of the paper will be accessible to readers.

For Research, Software, and Methods articles, you will receive an invoice from PLOS for your publication fee after your manuscript has reached the completed accept phase. If you receive an email requesting payment before acceptance or for any other service, this may be a phishing scheme. Learn how to identify phishing emails and protect your accounts at https://explore.plos.org/phishing.

Thank you again for supporting PLOS Computational Biology and open-access publishing. We are looking forward to publishing your work!

With kind regards,

Janani Seenivasan

PLOS Computational Biology | Carlyle House, Carlyle Road, Cambridge CB4 3DN | United Kingdom ploscompbiol@plos.org | Phone +44 (0) 1223-442824 | ploscompbiol.org | @PLOSCompBiol
